# Retrospective analysis of *Plasmodium vivax* genomes from a pre-elimination China inland population in the 2010s

**DOI:** 10.3389/fmicb.2023.1071689

**Published:** 2023-02-09

**Authors:** Ying Liu, Tao Zhang, Shen-Bo Chen, Yan-Bing Cui, Shu-Qi Wang, Hong-Wei Zhang, Hai-Mo Shen, Jun-Hu Chen

**Affiliations:** ^1^National Institute of Parasitic Diseases, Chinese Center for Diseases Control and Prevention (Chinese Center for Tropical Diseases Research), Shanghai, China; ^2^National Health Commission of the People’s Republic of China (NHC) Key Laboratory of Parasite and Vector Biology, Shanghai, China; ^3^World Health Organization (WHO) Collaborating Center for Tropical Diseases, Shanghai, China; ^4^National Center for International Research on Tropical Diseases, Shanghai, China; ^5^Henan Provincial Center for Disease Control and Prevention, Zhengzhou, China; ^6^Anhui Provincial Center for Disease Control and Prevention, Hefei, China; ^7^School of Global Health, Chinese Center for Tropical Diseases Research, Shanghai Jiao Tong University School of Medicine, Shanghai, China

**Keywords:** malaria, China inland, *Plasmodium vivax*, haplotype-based detecting, positive selection, drug resistance

## Abstract

**Introduction:**

In malaria-free countries, imported cases are challenging because interconnections with neighboring countries with higher transmission rates increase the risk of parasite reintroduction. Establishing a genetic database for rapidly identifying malaria importation or reintroduction is crucial in addressing these challenges. This study aimed to examine genomic epidemiology during the pre-elimination stage by retrospectively reporting whole-genome sequence variation of 10 *Plasmodium vivax* isolates from inland China.

**Methods:**

The samples were collected during the last few inland outbreaks from 2011 to 2012 when China implemented a malaria control plan. After next-generation sequencing, we completed a genetic analysis of the population, explored the geographic specificity of the samples, and examined clustering of selection pressures. We also scanned genes for signals of positive selection.

**Results:**

China’s inland populations were highly structured compared to the surrounding area, with a single potential ancestor. Additionally, we identified genes under selection and evaluated the selection pressure on drug-resistance genes. In the inland population, positive selection was detected in some critical gene families, including *sera, msp3*, and *vir*. Meanwhile, we identified selection signatures in drug resistance, such as *ugt, krs1*, and *crt*, and noticed that the ratio of wild-type *dhps* and *dhfr-ts* increased after China banned sulfadoxine-pyrimethamine (SP) for decades.

**Discussion:**

Our data provides an opportunity to investigate the molecular epidemiology of pre-elimination inland malaria populations, which exhibited lower selection pressure on invasion and immune evasion genes than neighbouring areas, but increased drug resistance in low transmission settings. Our results revealed that the inland population was severely fragmented with low relatedness among infections, despite a higher incidence of multiclonal infections, suggesting that superinfection or co-transmission events are rare in low-endemic circumstances. We identified selective signatures of resistance and found that the proportion of susceptible isolates fluctuated in response to the prohibition of specific drugs. This finding is consistent with the alterations in medication strategies during the malaria elimination campaign in inland China. Such findings could provide a genetic basis for future population studies, assessing changes in other pre-elimination countries.

## Introduction

*Plasmodium vivax* is the most widely distributed human malaria species, with an estimated 350 million Chinese individuals at risk and 30 million cases annually 7 decades ago (Yin et al., 2014). China was declared malaria-free by the World Health Organization in 2021, a remarkable achievement and the outcome of the national malaria program’s dedicated efforts (Cao et al., 2021). Despite significant progress in reducing the malaria burden, imported cases have increased due to human settlements and movement activities, cross-border migration, ecological changes, vector population dynamics, and multidrug resistance. Vulnerable populations, such as overseas workers and businessmen, acquire infections due to occupational exposure, including crop plantations, forestry, mining, development projects, and tourism.

Imported cases are especially challenging in malaria-free countries, as connections with neighboring countries with higher transmission rates increase the risk of parasite reintroduction. China reported its last local malaria case in 2016, 6 years after aiming for eradication, but imported malaria will remain a threat until global eradication is achieved (Feng et al., 2014; Lai et al., 2017). Although the Great Mekong Sub-region (GMS including Cambodia, China, Laos, Myanmar, Thailand, and Vietnam) has made significant progress in reducing transmission and is on track to achieve elimination by 2030, border surveillance will be necessary until the target is accomplished. Previous research has identified the risk of malaria transmission in China–Myanmar border areas and cross-border migration as a major source of the potential introduction of malaria into southern China (Zhou et al., 2014; Wang et al., 2015). This importation resulted from human activities involving border trade, businesses, and mass population movement due to political instability. Imported *P. vivax* cases have become a public health concern in malaria-endemic areas where the disease was eradicated many years ago. One of the most important lessons from the malaria elimination plan is to modify the strategy continuously as the malaria situation evolves and programmatic gaps are subsequently filled, allowing for more cost-efficient alternatives reflecting local transmission dynamics (Huang et al., 2022). Moreover, due to the COVID-19 pandemic, many countries have curtailed malaria elimination strategies. The key to addressing these challenges is the establishment of a genetic database for the rapid identification of malaria importation or reintroduction (Arnott et al., 2012). Several genome sequencing projects, such as MalariaGEN, have characterized the genetic diversity of *P. vivax* across populations. It also involves using novel technological advances such as sensitive and accurate genetic tools to trace the likely sources of malaria cases (Preston et al., 2014; Sturrock et al., 2015; Trimarsanto et al., 2019; Fola et al., 2020).

In this study, we retrospectively reported whole-genome sequence variation in 10 *P. vivax* isolates from inland China with high parasite density to provide a view of genomic epidemiology during the pre-elimination stage. Henan reported the last local infection in 2011, and Anhui reported the last two local infection cases in 2013 (our sampling occurred in 2012). Here, we indicate the number of cases in China from 2010 to 2015 to demonstrate the representativeness of our collection (Table 1). The samples were collected in the 2010s from the Anhui and Henan provinces and represented the last inland outbreaks before elimination. Our results revealed that the inland population was severely fragmented with low identity by descent (IBD) relatedness among infections, suggesting that super-infections or co-transmission events are rare in low-endemic areas. We also identified selection signatures in drug resistance and found that the ratio of sensitive isolates changed in response to restricted drugs.

**Table 1:** Reported malaria cases in China (with Anhui and Henan province) from 2010 to 2015.

Year	No. of cases	Suspected cases	Local cases	Vivax cases ratio (%)	Anhui		Henan	
					Total cases	Local cases	Total cases	Local cases
2010	7,855	34,082^*^	^*^	^*^	1864	^*^	893	^*^
2011	4,479	1,311	1,314	56.7	644	526	358	166^**^
2012	2,718	0	182	39.7	98	30^**^	151	0
2013	4,128	0	48	22.8	184	2	197	0
2014	3,078	0	56	27.7	144	0	216	0
2015	3,288	0	40	26.9	128	0	184	0

Data were downloaded from the following papers: (Zhou et al., 2011; Xia et al., 2012; Xia et al., 2013; Zhang et al., 2014; Zhang et al., 2015; Zhang et al., 2016).

* China has just promulgated the National Malaria Elimination Plan (NMEP) in 2010, so the previous data have not been fully recorded. In addition, because there was no immediate sampling system at that time, many samples were not retained, which led to many suspected cases.

** This number includes all types of malaria. We collected the local samples from here and used in this study.

## Materials and methods

### Ethics statement

The study was approved by the Ethics Committee of the National Institute of Parasitic Diseases (NIPD), Chinese Center for Disease Control and Prevention (China CDC). The participants were informed of the study’s procedure, potential risks, and benefits. After the participants agreed to participate in the study, written informed consent was obtained from them.

### Collection of genomic data

We used the 2016 genotype call data set (Variant Call Format file) with 228 samples from the following countries: Cambodia, China, India, Indonesia, Laos, Malaysia, Myanmar, Thailand, Vietnam, and Papua New Guinea (Pearson et al., 2016). Information, such as collection location and time, was downloaded from “www.malariagen.net/data/p-vivax-genome-variation-may-2016-data-release.” We included published genomic data of six clinical samples collected from the China–Myanmar border (CMB) area (Shen et al., 2017) in our reference dataset. Single-nucleotide polymorphism (SNP) information and allele frequencies were downloaded from the *P. vivax* Genome Variation Project, converted to the Vcftools 012 form, and then merged with our own data. In addition, annotation of the *SaL* I reference database was downloaded from PlasmoDB (Aurrecoechea et al., 2008). For a parallel analysis with PvP01 (Auburn et al., 2016) as mapping targets, we downloaded additional reference sequences from countries around the world for principal component analysis (PCA) and structure analysis: Myanmar (PRJNA603279; Brashear et al., 2020), Brazil (PRJNA240378-98), Columbia (PRJNA240414-44), Mexico (PRJNA240445-64), Peru (PRJNA240367-530), PNG (PRJNA240366-530), and Malagasy (PRJNA175266; Chan et al., 2012), the BioProject and SRA numbers of those samples are listed in the Appendix file (Supplementary Table S5).

### Sampling *Plasmodium vivax* parasites and genome sequencing

Blood samples were collected from 10 clinical malaria cases in the Anhui and Henan provinces in 2011–2012 that were microscopically positive and PCR-confirmed for a single *P. vivax* infection. Henan reported the last local case in the same year, while Anhui reported the last two local cases the following year. According to the National Malaria Elimination Plan (NMEP) promulgated in 2010, the provincial CDC must go to the hospital for sampling and PCR identification immediately after receiving the case report, which is also how we obtained the samples. Simultaneously, the CDC conducted a detailed on-site investigation to determine if the case was imported. Samples with high parasite density (more than 10,000 parasites/μl) in early records were selected to ensure sequencing integrity. We extracted *P. vivax* genomic DNA from unfiltered infected whole blood (the blood samples could not be filtered because they had been frozen with EDTA at −20°C for too long). We extracted more than 50 ng of total DNA from each sample and ensured that the concentration was greater than 1 ng/μl. Unfortunately, many samples degraded due to long storage times and failed to meet this requirement. Specifically, each frozen blood sample DNA was extracted using a QIAGEN DNeasy Blood & Tissue Kit (Qiagen, United Kingdom). Libraries were prepared using the FC-121-4001 TruSeq Nano DNA LT Sample Preparation Kit (Illumina, United States) and sheared into 500 bp fragments using a Covaris S2 Focus Ultrasonicator. Due to insufficient DNA content in the samples, we could not use the CF11 column for filtration but proceeded directly to sequencing. In a previous study (Chen et al., 2017), we confirmed that direct sequencing did not affect the data. All libraries on Illumina X-10 were sequenced, generating an average of 64 M (46–87 M) paired-end reads of 150 bp. Illumina sequencing raw reads are available in the NCBI Sequence Read Archive under the BioProject accession number PRJNA868867.

### Variant identification and filtering

Sequenced reads were filtered using Trimmomatic-3.0 (Bolger et al., 2014) to remove adapter and low-quality sequences and mapped to *P. vivax SaL* I genome using BWA (Li and Durbin, 2010). The Bam file was modified using Picard2.6 tools FixMateInformation and MarkDuplicates, and genotyping was performed using a base quality score recalibration pipeline based on GATK4 workflows (McKenna et al., 2010). We ran GATK BaseRecalibrator on each BAM file to generate a recalibration table based on various covariates and used known-site gvcf files published previously (Pearson et al., 2016) to establish base quality scores. Vcftools (Danecek et al., 2011) was used to remove indels and filter loci with a MAF of less than 0.05. A variable proportion of reads (8.52–12.6%) from all isolated samples were mapped to the reference genome and aligned to 90% of the reference genome with high fold coverage (12–28x). Excluding SNPs with >5% missing calls from high-quality samples yielded 184,401 high-quality SNPs. Missing calls were defined as positions with fewer than two reads.

We also conducted a parallel analysis using PvP01 as the mapping target. We found that the mapping ratio was lower, resulting in higher FWS and larger SNP differences. When P01 was used as a reference, the FWS values were all greater than 0.95. This suggested that all samples were single-lineage infections, which seemed inconsistent with the general pattern. Here, we exclusively present PCA and structure analysis using the P01 reference and additional reference sequences from around the world to demonstrate the uniqueness of samples from inland China.

### Population genetics and structure test

In this study, we restricted the population diversity and divergence analysis to heterozygous major allele calls. In an effort to maintain as many SNPs as possible, many low-quality SNPs were retained but not all of them were used. Only those SNPs of published loci were used for PCA, phylogenetic tree, and structural analysis. Total SNPs were used for selection pressure and single-gene analyses. The within-host infection complexity was assessed by within-sample F-statistic (F_WS_) values and calculated using the Moimix R package. Pairwise IBD was measured using hmmIBD with default parameters and program-estimated allele frequencies (Schaffner et al., 2018). The pairwise IBD outputs were then determined at 1 kb intervals across each chromosome, and the average fractions of IBD derived from each position in each population were calculated.

We evaluated the population structure using both the surrounding area sample and 10 high-coverage single-infection isolates. For high-quality SNP, we estimated the nucleotide diversity ($\hat{\pi }$ ), Watterson’s estimator ($\hat{\theta }$ _ω_), genetic differentiation (F_ST_), and Tajima’s D-value across 4,653 genes on 14 chromosomes in ARLEQUIN-Ver3.5 (Excoffier and Lischer, 2010). PCA was performed using the R package, and the neighbor-joining (NJ) tree was constructed using MEGA (Tamura et al., 2013). The ancestry shared by individual isolates was analyzed using ADMIXTURE software (Alexander et al., 2009), and the optimal K cluster value was determined by running the software multiple times with different *K*-values.

### Positive selection tests

The integrated haplotype score (iHS) and cross-population extended haplotype homozygosity (XP-EHH) were used to detect recent or ongoing positive selection in Selscan-Ver1.10a (Szpiech and Hernandez, 2014). These statistical analyses are based on the selective sweep model where mutation occurs in a haplotype and quickly sweeps toward fixation, reducing the locus diversity. Integrated haplotype score (iHS) is the standardized log ratio of the integrated extended haplotype homozygosity (EHH), which is calculated by tracking the decay of haplotype homozygosity for all ancestral and derived haplotypes extending from each SNP site (Sabeti et al., 2002; Voight et al., 2006). SNP with inferred ancestral states and minor allele frequencies of at least 5% were used for iHS. Each raw score was normalized to genome-wide frequency bins. During the EHH computation of each SNP locus, if the start/end of a chromosome arm was reached before EHH <0.05 or if a physical distance (kbp) between two markers >200 was encountered, the calculation was aborted. Cross-population extended haplotype homozygosity (XP-EHH) is the standardized log ratio of the integrated site-specific EHH at core SNP between two populations, which in this study were defined as China inland samples and reference samples (Sabeti et al., 2007). Site-specific EHH does not require polymorphic markers within the population. Therefore, it can detect selective sweeps for alleles that have undergone fixation. In our calculation, the sums in each locus were truncated at the SNP with an EHH value of <0.05 or if the computation extended more than 1 Mbp from the core loci. Previous analyses have suggested that iHS has the maximum capacity to detect selective sweeps that have reached moderate frequency, while XP-EHH can detect selective sweeps at high frequency, thus making the two tests complementary.

## Results

### Genomic data summary

A total of 10 samples had high-quality genomic data and generated between 46 and 87 million paired-end reads, with an average read length of 150 bp. A variable proportion of reads (8.52–12.6%) were mapped to the *P. vivax SaL* I reference. The average coverage of the entire genome by the filtered consensus base calls is 93.16%, and 94.18% of chromosomes were generated (Table 2).

**Table 2:** Sequencing and mapping summary statistics for 10 China inland samples.

Sample ID	Total reads	Mapped ratio (%)	Average coverage depth (X)	Genome coverage >1x (%)	Covrage >10x (%)	F_WS_
M001B	85,817,050	8.89	19.71	94.45	8.38	0.9264
M052B	87,590,464	8.52	28.18	93.70	5.27	0.9398
S2011054	55,480,824	9.80	12.85	92.00	2.42	0.9861
S2011067	83,067,520	8.69	16.42	92.59	4.67	0.9492
S2011068	60,557,874	10.37	15.31	91.81	7.76	0.9056
S2011069	5,3,677,630	10.27	13.87	92.26	7.33	0.9119
S2011097	59,224,092	12.63	22.04	96.21	59.21	0.9005
Sample_225	52,169,688	9.68	12.48	90.48	4.52	0.9478
Sample_364	55,316,880	10.52	16.49	95.78	20.93	0.8569
Sample_455	46,641,940	11.92	13.29	92.36	7.09	0.9273

### Population structure of China inland samples

**Figure 1:**
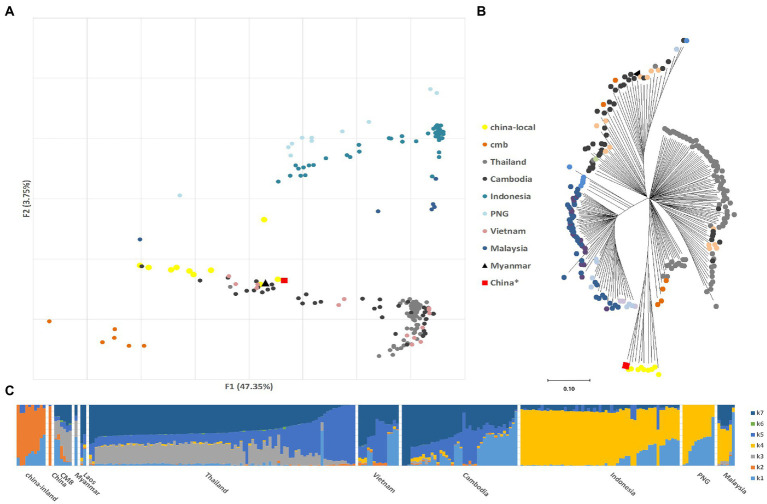
Parasite population structure in China inland samples relative to the reference (Cambodia, China*, India, Indonesia, Laos, Malaysia, Myanmar, Thailand, Vietnam, and Papua New Guinea). The China* samples (SAMEA2358527) are also downloaded from the study of Pearson et al. (2016), which was collected from the Yunnan border area, China in 2011, and published on ENA in 2014. **(A)** Principal component analysis (PCA) plots illustrating the genetic differentiation between populations; **(B)** Neighbor-joining tree illustrating the relatedness between the inland *Plasmodium vivax* isolates relative to the reference with 1,000 bootstraps. **(C)** ADMIXTURE bar plot illustrates the population structure within and among populations at an optimized cluster value of *K* = 7.

The population structure was investigated using PCA and a phylogenetic tree of SNP variations. *Plasmodium vivax* clustered according to their geographic origin, and the Pacific island samples could be distinguished from continental samples (Figure 1A). The major components constituted a distinct inland cluster in China, which was coordinated by a Chinese reference (Pearson et al., 2016). These differences were also evident in the NJ tree (Figure 1B), which divided Asia (Thailand, Myanmar, Cambodia, Vietnam, and China) from the Pacific Islands (Malaysia, Indonesia, and Papua New Guinea). Unexpectedly, eastern Southeast Asian samples (Cambodia and Vietnam) exhibited greater genetic relatedness and were separated by CMB samples. Parallel analysis using PvP01 as a reference revealed substantial differences between the South American and Asian samples in the PCA test (Supplementary Figure S1), confirming that geographic location remains a primary determinant of variance.

The ADMIXTURE analysis identified several major populations that corresponded to Asian samples (Figure 1C). The GMS group, including CMB, resembled a mixture of the Southeast Asian components K7, K5, and K3. K4 represented Pacific island samples and was rare in continental populations. K2 only appeared in the Chinese and CMB groups. In other words, K2 and K4 were highly structured populations with shared ancestry that overcame long geological isolation. China is technically not a Southeast Asian country, and K3 was special and only identified in Thailand and CMB samples, indicating that the complex structure of border malaria was caused by transmission and mixing from both sides. In the parallel structure test with PvP01 strain as the reference, we found (Supplementary Figure S2) that the inland Chinese populations belong to the same potential ancestral taxonomy and are quite different from the Yunnan-collected Chinese border samples.

**Figure 2:**
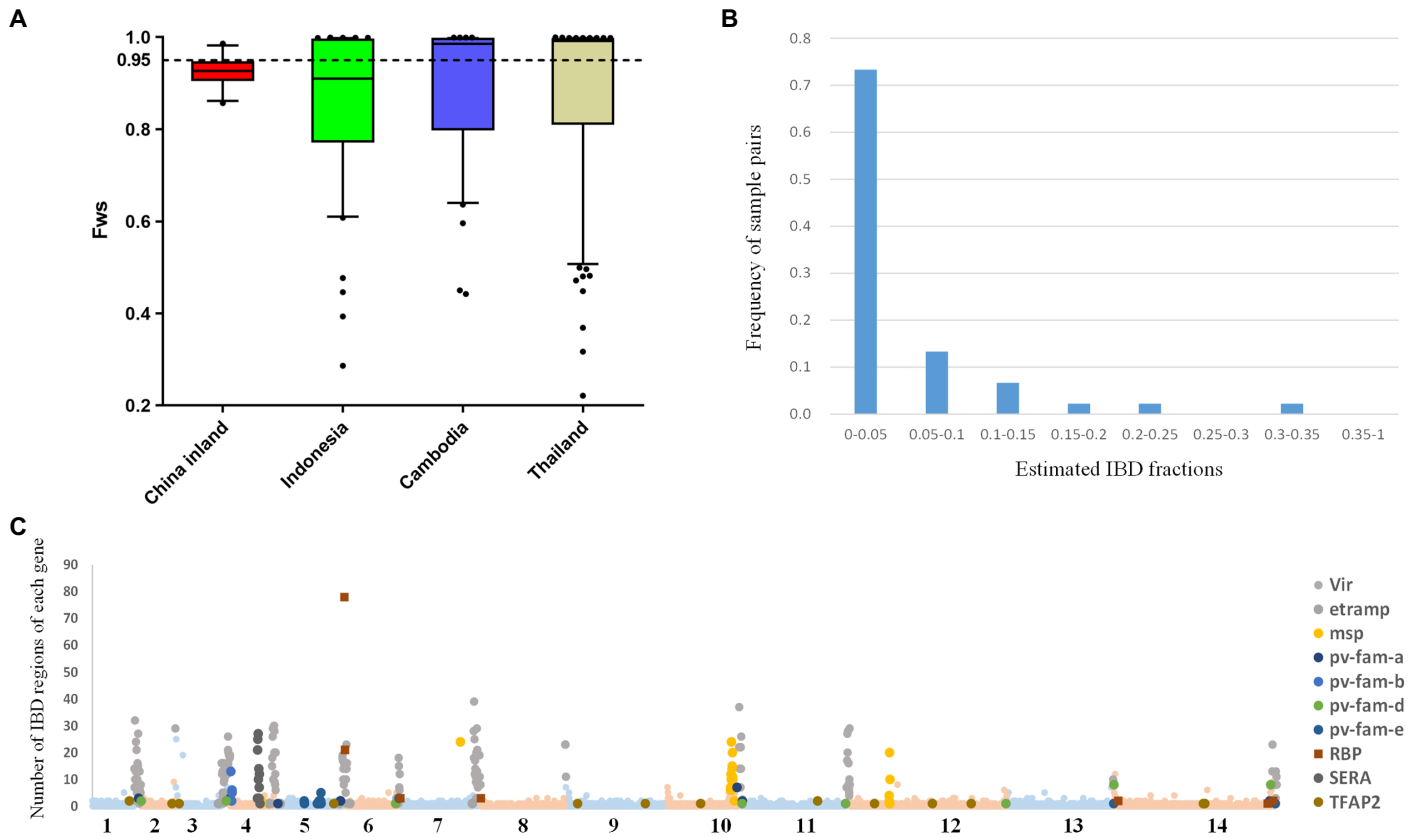
F_WS_ and IBD plots illustrate the complexity and relatedness among infections in China’s inland population. **(A)** Boxplots illustrating different trends in inland, Indonesia, Cambodia, and Thailand. The dotted line illustrates the F_WS_ = 0.95, above which infections are essentially monoclonal. **(B)** Patterns of identity by descent (IBD) were explored across the genome of China inland and reference populations. The histogram represents the frequency of sample pairs, each pair with IBD fractions above 0.5 should be considered related. **(C)** 3,431 IBD regions were found and involved 1,157 genes, and 123 genes containing more than 10 IBD fragments including members of multigene families. The *x*-axis of the scatterplot represents the 14 chromosomes of the parasite and the *y*-axis represents the number of IBD regions for each gene.

We calculated within-sample parasite diversity in inland Chinese samples to investigate the complexity of infection. In these samples, *F*_WS_ values of individual infections ranged from 0.85 to 0.98 (mean 0.925 and median 0.927), whereas, in Thailand and Indonesia, values ranged from 0.22 to 0.99 (mean 0.87 and median 0.99) and from 0.28 to 0.99 (mean 0.85 and median 0.91), respectively. An *F*_WS_ value of 0.95 indicates that an infection predominantly contains a single genotype, even if additional genotypes are present at relatively low proportions. The *F*_WS_ values revealed a lower proportion of monoclonal infections in the China inland sample (3/10) compared with Thailand (59.78%, 55/92) and other populations (Figure 2A); however, this difference was not statistically significant due to the small sample size. For each segregating allele locus across the low *F*_WS_ value samples, most of these positions had >5X read depth in major calls but insufficient depth in minority calls, implying that the risk of unrelated parasites from separate mosquito bites was quite low. Without follow-up reports of relapses, it is evident that these cases have neither relapsed nor recurred and that genetic complexity is unlikely to affect the genetic analysis process.

Meanwhile, we checked the chromosome-level structure of inland Chinese isolates with the identity by descent (IBD) fraction, which is widely used to study relatedness among proximal parasite populations (Figure 2B). Although inland China samples exhibited the highest fractions of pairwise IBD (mean value = 0.059 in CMB) compared to any other GMS population (0.016 in Cambodia and 0.019 in Thailand) from previous research, the IBD values were still low. This was probably because the sample size was inadequate. In the inland Chinese population, 3,431 IBD regions involving 1,157 genes were identified. Among them, 123 genes contained more than 10 IBD fragments (Figure 2C), including multigene family members, such as *msp*, *sera*, and *vir*. Remarkably, the gene with the most IBD fragments is the reticulocyte-binding protein (*pvrbp2c*, PVX_090325), which is well-known for its erythrocyte-binding-related conservative regions (Gupta et al., 2018).

### Genomic scan for differentiation between inland and border populations

To explore the genomic profile of divergence among *P. vivax* populations, we estimated the fixation index (F_ST_) of individual genes for the populations separated by differing geographical distances (Supplementary Table S1). Not surprisingly, the Indonesian and Chinese inland populations exhibited considerable differentiation for each gene (mean F_ST_ = 0.26, median 0.22), which could be attributed to geographic variations between the island and continental samples. In contrast, the average differentiation for each gene in inland China and border samples was lower (mean F_ST_ = 0.18, median 0.15), and 339 genes had F_ST_ values of >0.5. Gene ontology (GO) term analysis was conducted among these genes to assess which functions were enriched. Genes associated with an intracellular anatomical structure (GO:0005622), RNA binding (GO:0003723), organelles (GO:0043226), and nitrogen compound biosynthetic processes (GO:1901564, GO:0006807, and GO:0044271) were found to be significantly enriched.

### Identifying signatures of selection in CMB

**Figure 3:**
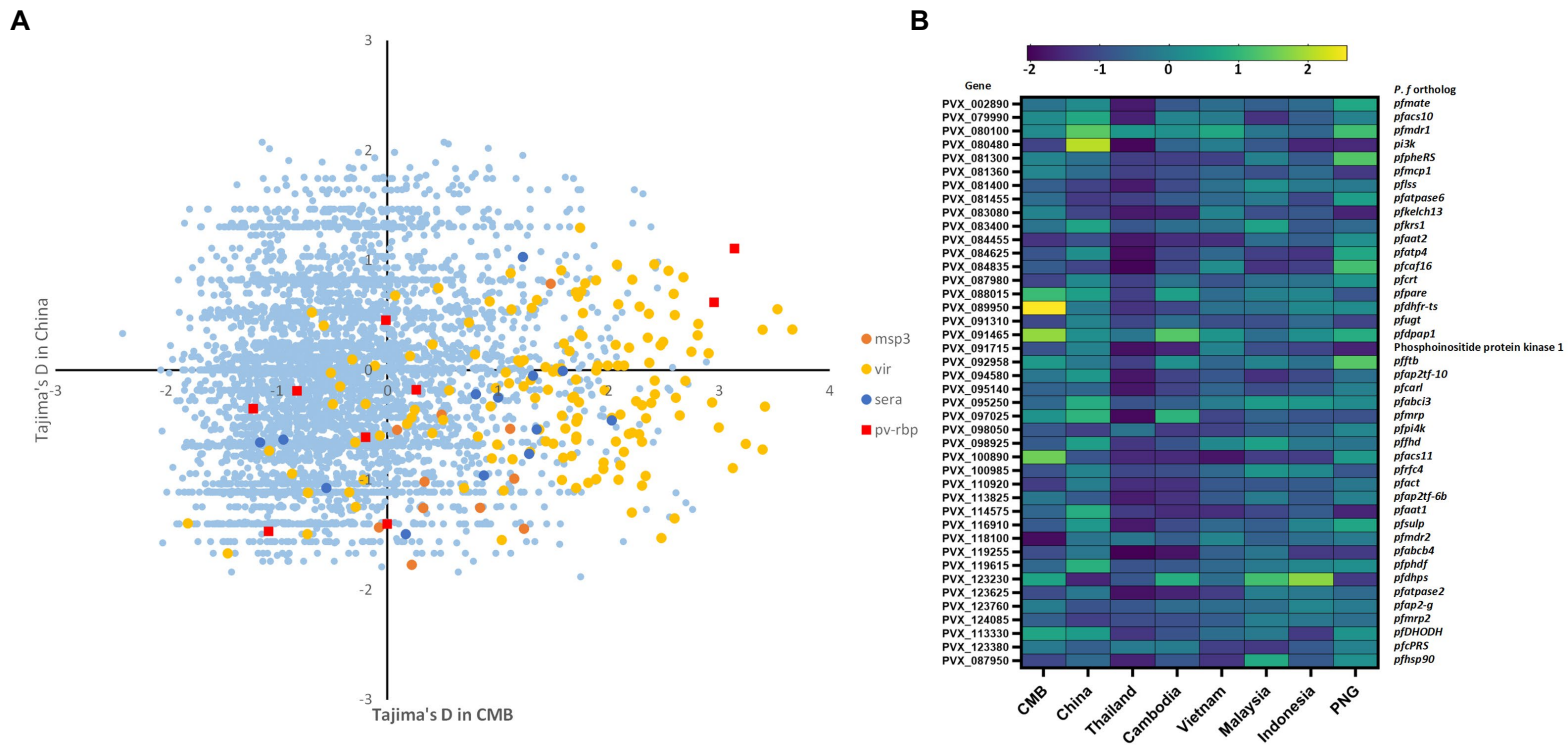
Balanced selection (Tajima’s D-value) of 5,249 genes in inland and border populations. **(A)** Balanced selection (Tajima’s D-value) of all genes in both populations. We selected some immune-related genes such as *vir*, *msp.*, and *rbp* to evaluate the differences in environmental stress between China inland and CMB samples. Tajima’s D-values of China inland samples were mostly negative with a mean of −0.12, a bit higher than CMB. However, there was a clear difference in *vir* gene families, consistent with the difference in malaria transmission between the two places in the 2010s. **(B)** A balanced selection of 42 orthologs of *P. falciparum* drug-resistance genes summarizing the difference between inland and reference populations. The Southeast Asian population exhibited higher overall pressure levels than Pacific island populations in Tajima’s D-test but no significant difference in *pvcrt* and *pvmdr1*. In antifolate-resistance genes, there are positive balance selective pressures in the Southeast Asian population but negative balance selective in island populations.

The analysis focused on the 4,624 genes with at least three SNP each to examine allele frequency distributions for specific genes in the Chinese inland population. Tajima’s D-values of inland samples were mostly negative, with a mean of −0.12, which was slightly higher than that of CMB (mean = −0.35 and medium = −0.48); 2,083 genes (27%) had positive Tajima’s D-values (Figure 3A). Tajima’s D value of 42 orthologs of P. falciparum drug-resistance genes were summarized to reveal the difference between inland and reference populations (Figure 3B). In the other Southeast Asia (SEA) reference population, Thailand samples had 4,865 genes with at least three SNPs, a mean of −1.61, a median of −1.72, and only 88 genes (1.8%) had positive Tajima’s D-values. However, there was a clear distinction between *vir* gene families (the average for *vir* was −0.06 in inland and 1.13 in the CMB group separately). This difference corresponded with the disparity in malaria transmission between the two locations in the 2010s. In contrast to the PCA result, the balancing selection of genes did not show consistency in geographic origin. Here, we used Tajima’s D-test to distinguish between genes evolving neutrally or during the selection process. The higher the number of genes subject to negative balance selection, the greater the environmental pressure on the population, just as the selection pressure on drug resistance genes can reflect alterations in local drug use strategies. Both belong to the Pacific Islands population, and 4,657 genes with at least three SNPs and 508 genes (10.9%) with positive Tajima’s D-values were included in our calculation for the Indonesian population. However, 4,232 available genes in PNG were included, and 1,284 (30%) had positive values. Consistent with our prior results, the predominantly negative values in SEA isolates indicate a recent population expansion of *P. vivax* in the CMB area (Shen et al., 2018). Compared to highly endemic populations, such as Thailand, inland China samples exhibited a different environment with a lower selection pressure.

### Positive selection tests

**Figure 4:**
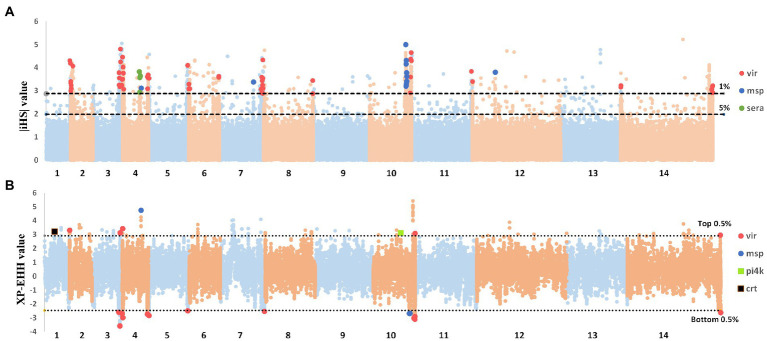
Genome-wide scans to identify regions with extended homozygosity using haplotype-based detecting positive selection. **(A)** Genome-wide scan of standardized |iHS| for *Plasmodium vivax* SNPs with a minor allele frequency of at least 5% in inland samples. Dashed lines indicate the top 1% of |iHS| values (|iHS| score of >2.8). **(B)** Genome-wide scan of standardized XP-EHH for *P. vivax* SNPs with a minor allele frequency of at least 5% in inland samples. Using CMB isolates as the reference population. Dashed lines indicate the top 1% of XP-EHH values (±0.5%, XP-EHH score of >0.78 or < −0.24 in CMB-China).

This study used the integrated haplotype score (iHS) statistic to detect incomplete sweeps and cross-population extended haplotype homozygosity (XP-EHH) in cases where the sweep was near fixation within the population. Using the |iHS| score for all SNPs (MAF of >5%) in the CMB samples, we identified 14 chromosomal regions with SNP values above the top 5% of the randomly expected distribution (Supplementary Table S2). Our previous analyses revealed that positively selected SNP loci are typically associated with genes for red blood cell invasion and immune evasion. The top 1% SNP loci included 127 genes (Figure 4A) whose functions were enriched with membrane-intrinsic components (GO:0031224) and cysteine-type peptidase activity (GO:0008234). Elevated |iHS| values were observed in some important gene families, such as *sera* (four genes, mean |iHS| = 3.49), *msp3* (nine genes, mean |iHS| = 3.7), *vir* (58 genes, mean |iHS| = 3.52), and *Pv-fam-d* (two genes, mean |iHS| = 4.32). The observation of positive selection is similar to the results obtained in previous studies, and the selection of vaccines targeting polymorphic antigens may explain the difficulty in eliciting cross-protective immune responses. The XP-EHH test was applied to compare the average haplotype length of each SNP between inland samples and the CMB references (Supplementary Table S3). It identifies areas in the genome where destination samples show much longer haplotypes than the reference, indicating recent positive selection in the tested population. These selection signals were stronger in CMB samples and included known marker resistance to chloroquine (*crt*) and artemisinin (*pi4k*; Park et al., 2012; Cerqueira et al., 2017). In contrast, only the *vir* and *msp3* gene families exhibited the most positive selection signals in inland isolates (Figure 4B).

### Genes associated with drug resistance in the inland isolate

During 2009–2016, molecular surveillance of drug-resistant *Plasmodium vivax* malaria identified chloroquine (CQ) resistance markers in southern and central Myanmar and higher mutation rates of antifolate resistance markers (Nyunt et al., 2017). In this study, we integrated a list of putative drug-resistance genes from earlier research (Gamo et al., 2010; Price et al., 2012; Hupalo et al., 2016; Plouffe et al., 2016) and compared these genes from different sources (Table 3; Supplementary Table S4). In Tajima’s D-test, the Southeast Asian population was predicted to exhibit higher overall pressure levels than the Pacific island populations (Figure 3B); however, there was no significant difference in the CQ resistance genes (*pvcrt* and *pvmdr1*). For antifolate resistance genes, we found positive and negative balance selective pressures in Southeast Asian and island populations, respectively, which could be related to the suspension of these drugs in Southeast Asia for almost 20 years. The disparities between China’s inland and reference populations were then analyzed. Some genes, such as chloroquine resistance transporter (*crt*), multidrug resistance (*mdr1*), and imidazole piperazine resistance, such as *ugt* and *krs1* (Lim et al., 2016; Saint-Léger et al., 2016), showed higher Tajima’s D-values in inland populations but lower in CMB, whereas genes related to sulfadoxine–pyrimethamine (SP) resistance, such as *dhps* and *dhfr-ts*, (Olliaro and Mussano, 2003) were under positive balance selection in the rest of the region.

**Table 3:** Putative *Plasmodium falciparum* drug resistance gene orthologous with their associated population genetic statistics.

Gene	Chr	$\hat{\pi }$	Tajima’s D value				Top 1% |ihs| value	Orthology in
			China inland	CMB	Thailand	PNG		*P. falciparum*
PVX_087980	1	1.01E−03	0.0974	−1.1107	−1.1584	0.3533	1.3021	*crt*
PVX_097025	2	5.02E−04	0.9345	0.3144	−1.9495	−0.9077	1.3703	*mrp*
PVX_089950	5	3.56E−04	−0.1839	2.5562	−1.3701	0.1677	0.2814	*dhfr-ts*
PVX_094580	8	4.66E−04	0.3764	−0.2311	−1.793	−0.373	1.3439	*ap2tf-10*
PVX_091310	9	0.00E+00	0	−1.0365	−1.0863	−1.1285	0.2381	*ugt*
PVX_079990	10	1.20E−03	0.7178	0.1391	−1.6328	−0.0249	0.8844	*acs10*
PVX_080100	10	8.50E−04	1.3814	0.1361	0.365	1.1506	1.6588	*mdr1*
PVX_080480	10	4.08E−04	2.0741	−1.1305	−1.9775	−1.5284	1.1136	*pi3k*
PVX_098050	10	5.23E−04	−1.1766	−0.7887	−0.3258	0.0362	1.439	*pi4k*
PVX_113825	11	3.21E−04	−0.7435	−0.2512	−1.6984	−0.0696	2.1363	*ap2tf-6b*
PVX_113330	11	9.18E−04	0.4449	0.6474	−1.3299	0.3358	0.873	*DHODH*
PVX_083400	12	5.67E−04	0.6242	−0.3317	−0.8159	−1.5999	0.9967	*krs1*
PVX_083080	12	9.35E−05	−1.1117	0	−1.7332	−1.6		*K13*
PVX_118100	12	6.87E−04	−0.2806	−1.9023	−0.2545	−0.1617	1.1937	*mdr2*
PVX_123230	14	5.40E−04	−1.5729	0.6293	−0.8105	−1.2645		*dhps*
PVX_100890	14	7.48E−04	−0.8427	1.5364	−1.5308	0.4126	0.8281	*acs11*
PVX_124085	14	2.50E−04	−1.1895	−0.6429	−0.9771	−0.3874	2.0798	*mrp2*

## Discussion

Importing malaria to countries where it has been eradicated is a major barrier to global elimination (Sturrock et al., 2015; Song et al., 2018). The spread of malaria in endemic nations has contributed to the development of drug resistance and hampered long-term eradication goals. Most malaria cases in elimination-stage countries are imported, posing a risk of re-establishing transmission in receptive areas (Hai-Juan et al., 2019). Even in malaria-free countries, imported cases often lead to delays in diagnosis, higher treatment costs, and local secondary transmission.

Recently, the number of imported cases in China has increased dramatically. From 2011 to 2015, 17,745 malaria cases were reported in mainland China; however, only 1,905 (11%) were locally transmitted (Zhou et al., 2016). As overseas investment from China continues to rise, the risk of malaria importation using returning laborers undertaking high-risk outdoor activities without proper protection increases. Parasite diversity is a fundamental predictor of transmission and immunity (Volkman et al., 2007; Manske et al., 2012). An understanding of the parasite population’s genetic structure is necessary to comprehend the epidemiology, diversity, distribution, and dynamics of natural *P. vivax* populations. Moreover, studying the population structure of genes under immune selection also reveals the dynamic interplay between transmission and immunity, which is crucial for vaccine development (Neafsey et al., 2012; Batista et al., 2015; Jennison et al., 2015). Therefore, this study aimed to retrospectively identify China’s inland malarial population. The genome data provide a unique opportunity to create SNP barcodes with a high tracing ability to identify parasite origin and a unique endemic setting for evaluating the dynamic shifts in transmission and selective pressures during the pre-elimination phase.

Our samples were collected from the last few inland outbreaks from 2011 to 2012 when China initiated a plan to control malaria transmission. Regarding population structure, China inland samples were clustered according to their geographic origin, continental samples differed from Pacific island samples, and the China–Myanmar border (CMB) samples could be regarded as a mixture of China and Myanmar samples with distinct characteristics from both countries. We found that representative sub-populations such as K2 closely matched the published Chinese reference. We observed low fractions of pairwise IBD values in the chromosome-level structure of inland samples, indicating that each infection occurred independently in a low-endemic setting. We conclude that our findings are broadly consistent with the assumptions for a pre-elimination population, except for the monoclonal infection ratio. The high incidence of mosquito bites during the outbreak could explain the lower monoclonal infections than in high transmission areas. Normally, a higher proportion of polyclonal infection reflects more frequent superinfection or co-transmission, which is rarely seen in low-endemic areas (Auburn et al., 2018). Conversely, a high ratio of monoclonal infections in small enclosed spaces such as islands could be due to a low entomological inoculation rate (EIR) or clonal parasite population. For example, recent research from the northeastern Peruvian Amazon revealed that the parasite population showed a high monoclonal ratio and diversity, inconsistent with geographical clustering, reflecting gene flow resulting from frequent travel in this area (Cowell et al., 2018).

Only the most drug-resistant infections survive in the pre-elimination environment, leading to intense drug selection (Maude et al., 2009). The rapidly shrinking population and low transmission rates may foster the emergence of multigenic resistance phenotypes. We suspected that stronger pressure signals could be detected in inland China samples and that the resistance alleles would be common and unlikely to be separated during recombination. Due to the high ratio of co-endemic *Plasmodium falciparum* in China, various artemisinin-based combination therapy (ACT) drugs have been used to shape genetic makeup (Douglas et al., 2010; Price et al., 2011). In our results, we observed selection signals for artemisinin resistance genes orthologous to *pi4k* (|iHS| = 1.44, Tajima’s D -1.17) and *atpase6* (|iHS| = 0.78, Tajima’s D -1.27). The XP-EHH results also indicated that *pi4k* was under stronger pressure in the CMB than in China. This is a global issue in GMS countries, as migrants may introduce drug-resistant strains to new locations. Meanwhile, Tajima’s D-test indicated a balanced selection of the most important drug-resistance genes, such as *crt* and *mdr1*. The data did not confirm our predictions of extended haplotype homozygosity for drug-resistance genes in the top 1% list.

Tajima’s D-test revealed that directional selection operating on *dhfr-ts* in China was significantly lower than in Thailand and Cambodia. This finding was expected because SP is only occasionally administered as an intermittent prophylactic treatment for pregnant women. However, we observed stronger pressure on *dhps* than in other GMS countries. We counted the frequency of *dhps* (amino acids 382, 383, 553, and 647) mutations and compared our findings with those reported previously (Na et al., 2005; Lu et al., 2010). We found a higher wild-type ratio, substantially lower than those from the 1990s but consistent with the studies from the 2010s. The remaining infections are drug-resistant, making eradication extremely challenging (Maude et al., 2009). For example, the annual use of ACT increases the proportion of artemisinin-resistant infections.

## Conclusion

A genetic database for the rapid identification of any importation or reintroduction of malaria is a critical step in addressing the worldwide and complex challenge of malaria eradication. We retrospectively reported whole-genome sequence variations in 10 *P. vivax* Chinese inland isolates from high parasite density regions to highlight the relevance of genomic epidemiology in the pre-elimination stage. Our results revealed that the inland population was severely fragmented with low relatedness among infections, despite a higher incidence of multiclonal infections, suggesting that superinfection or co-transmission events are rare in low-endemic circumstances. We identified selective signatures of resistance and found that the proportion of susceptible isolates fluctuated in response to the prohibition of specific drugs. This finding is consistent with the alterations in medication strategies during the malaria elimination campaign in inland China. This suggests that *P. vivax* China inland population may face more pressure to survive than around region in the 2010s.

## Data availability statement

Illumina sequencing raw reads described here are available at NCBI under accession number PRJNA868867.

## Ethics statement

This study was conducted according to the principles expressed in the Declaration of Helsinki. The study protocol, as well as potential risks and benefits, were explained to participants before blood collection, and all adult participants, as well as the parents or legal guardians of children, provided written informed consent. Blood was collected following institutional ethical guidelines reviewed and approved by the Ethics Committee of the National Institute of Parasitic Diseases, Chinese Center for Disease Control and Prevention.

## Author contributions

YL, TZ, H-MS, and J-HC conceived and designed the experiments and drafted the manuscript. S-BC and Y-BC conducted the experiments. YL and H-MS analyzed the data. TZ, S-BC, and Y-BC contributed to the reagents, materials, and analysis tools. All authors contributed to the article and approved the submitted version.

## Funding

This study was supported by the Project of the Shanghai Science and Technology Commission (Grant No. 18490741100), the Shanghai Municipal Health Commission Planning (Grant No. 201840007), the National Sharing Service Platform for Parasite Resources (Grant No. TDRC-2019-194-30), the Foundation of National Science and Technology Major Program (Grant No. 2012ZX10004-220), and the Open Project of NHC Key Laboratory of Parasite and Vector Biology (Grant No. WSBKFKT2019-03). The funding bodies had no role in the design of the study, in the collection, analysis, and interpretation of data, or in writing the manuscript.

## Conflict of interest

The authors declare that the research was conducted in the absence of any commercial or financial relationships that could be construed as a potential conflict of interest.

## Publisher’s note

All claims expressed in this article are solely those of the authors and do not necessarily represent those of their affiliated organizations, or those of the publisher, the editors and the reviewers. Any product that may be evaluated in this article, or claim that may be made by its manufacturer, is not guaranteed or endorsed by the publisher.

## Supplementary material

Supplementary Figure S1 PCA plots illustrating the parasite population structure in China inland samples relative to reference mapped with the P01 genome. The China* samples are also downloaded from Pearson et al.’s study, which was collected from Yunnan, China in 2011.

Supplementary Figure S2 ADMIXTURE structure in China inland samples relative to the reference mapped with the P01 genome. The China* samples are also downloaded from Pearson et al.’s study, which was collected from Yunnan, China in 2011. The ADMIXTURE bar plot illustrates the population structure within and among populations at an optimized cluster value of *K* = 5.
