# Supplementary figures and images for: Retrospective analysis of Plasmodium vivax genomes from a pre-elimination China inland population in the 2010s
Source: Front Microbiol. 2023 Feb 9;14:1071689. doi: 10.3389/fmicb.2023.1071689 (PMC9948256; doi:10.3389/fmicb.2023.1071689)

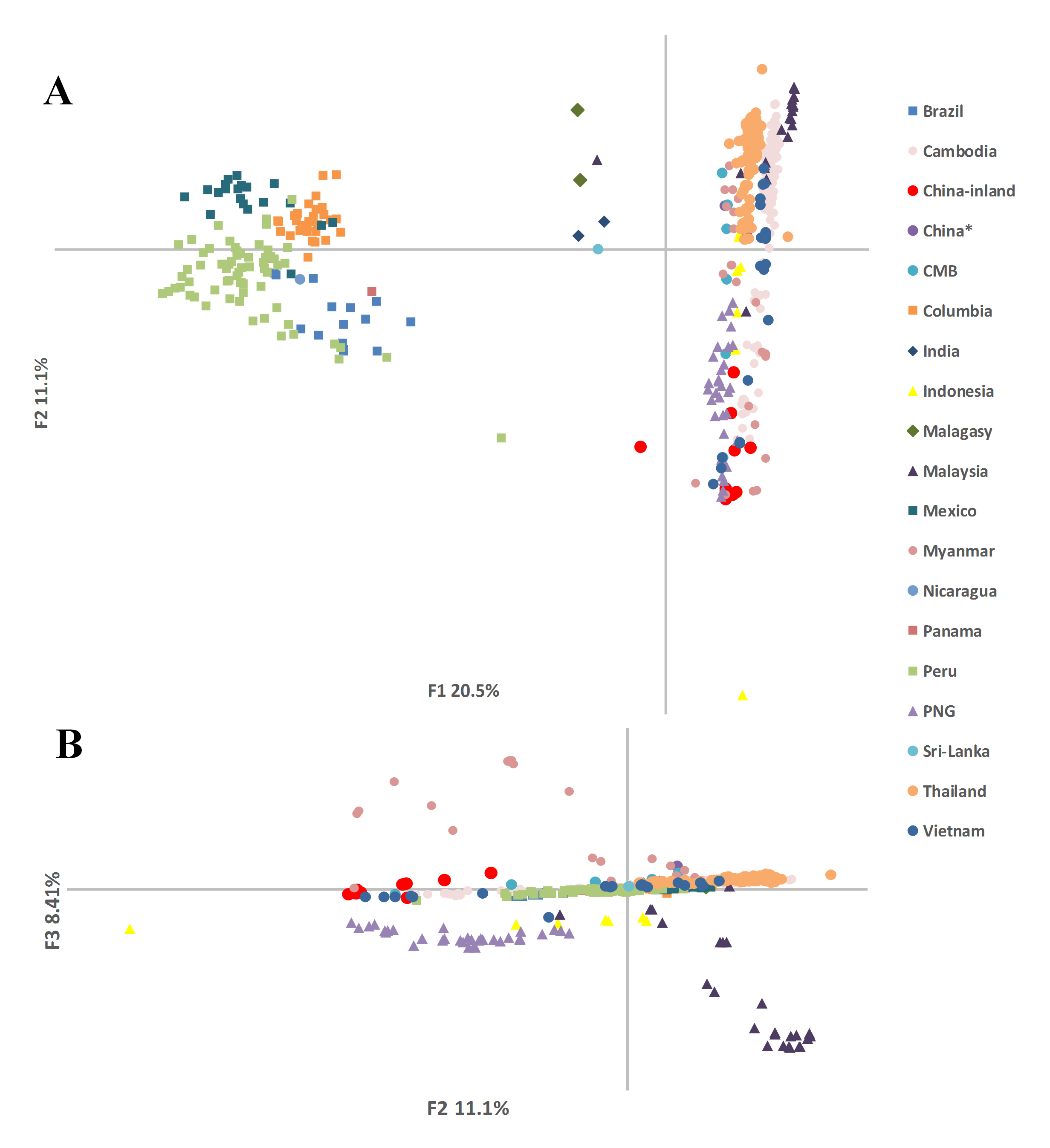

Supplement: Supplementary Figure S1 — PCA plots illustrating the parasite population structure in China inland samples relative to reference mapped with the P01 genome. The China* samples are also downloaded from Pearson et al.’s study, which was collected from Yunnan, China in 2011. [file Image_1.TIF]

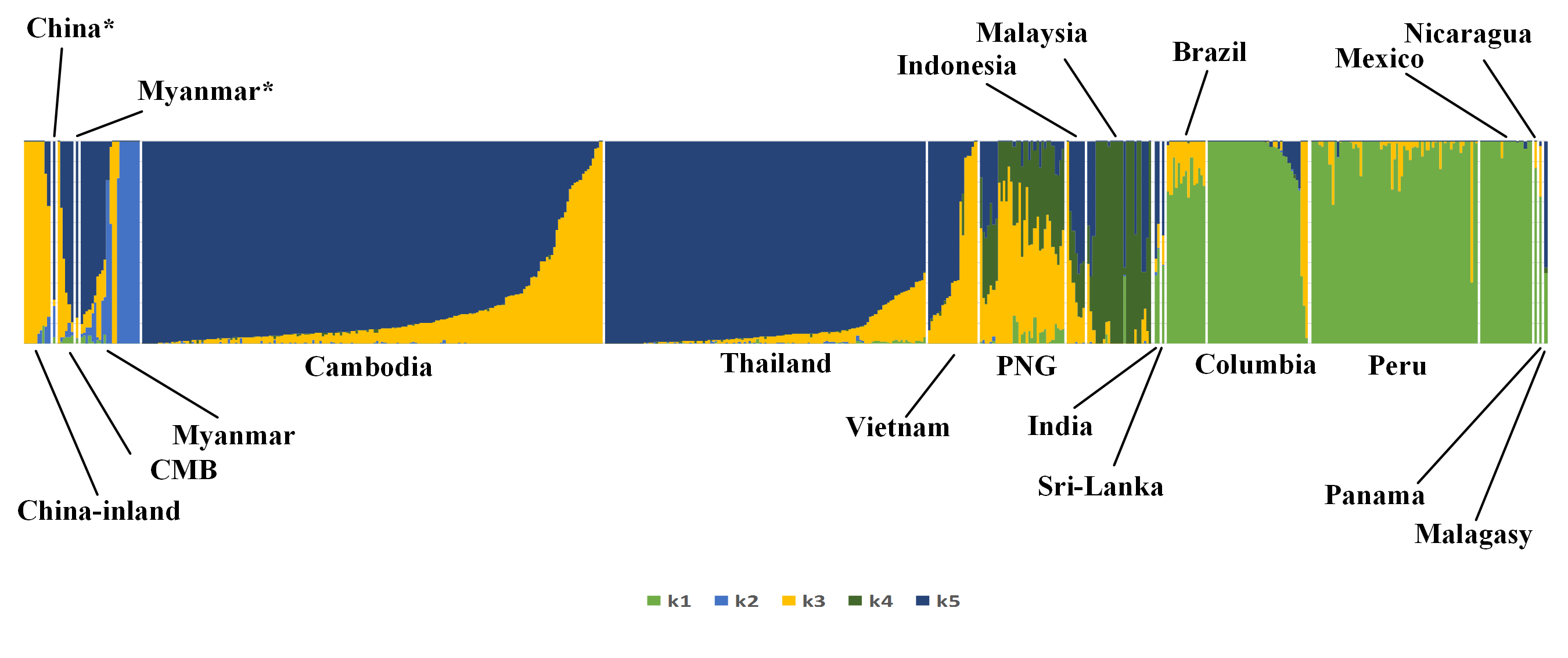

Supplement: Supplementary Figure S2 — ADMIXTURE structure in China inland samples relative to the reference mapped with the P01 genome. The China* samples are also downloaded from Pearson et al.’s study, which was collected from Yunnan, China in 2011. The ADMIXTURE bar plot illustrates the population structure within and among populations at an optimized cluster value of K = 5. [file Image_2.TIF]
